# First Replication of the Involvement of *OTUD6B* in Intellectual Disability Syndrome With Seizures and Dysmorphic Features

**DOI:** 10.3389/fgene.2018.00464

**Published:** 2018-10-10

**Authors:** Letizia Straniero, Valeria Rimoldi, Giulia Soldà, Melissa Bellini, Giacomo Biasucci, Rosanna Asselta, Stefano Duga

**Affiliations:** ^1^Humanitas Clinical and Research Center, Rozzano, Italy; ^2^Department of Biomedical Sciences, Humanitas University, Pieve Emanuele, Italy; ^3^Department of Pediatrics and Neonatology, Guglielmo da Saliceto Hospital, Piacenza, Italy

**Keywords:** intellectual disability, seizures, dysmorphism, OTUD6B, deubiquitinase, splicing mutation

## Abstract

Biallelic mutations in the ovarian tumor domain-containing 6B (*OTUD6B*) gene, coding for a deubiquitinating enzyme, were recently described to cause an intellectual disability syndrome characterized by seizures and dysmorphic features in six families worldwide. We here report on a 6-year-old Italian girl, presenting mild intellectual disability, speech and motor delay, and recurrent seizures, who came to our attention after being screened for genes responsible for Rubinstein–Taybi syndrome, Kabuki syndrome, and epilepsy. We hence submitted the proband’s DNA to whole-exome sequencing, disclosing two candidate heterozygous splicing mutations in the *OTUD6B* gene: c.324+1G>C and c.405+1G>A. Both variants are reported in the GnomAD database with a frequency lower than the 10^−5^ and affect the donor splicing site, of exons 2 and 3, respectively. Sanger sequencing confirmed the segregation of the variants in the family, showing that both parents are carriers of one mutation. RT-PCR experiments demonstrated that both variants affect *OTUD6B* splicing and lead to the production of aberrant transcripts, the major ones being, in both cases, the skipping of the upstream exon. Quantitative analysis performed by competitive-fluorescent RT-PCR on the patient RNA showed that the proband presents less than 1% of wild-type transcripts, further strengthening the causative role of these variants. This represents the first replication of the involvement of the *OTUD6B* gene in this syndrome and points to the appropriateness of screening *OTUD6B* in suspected Rubinstein–Taybi syndrome patients with negative results after the screening of the major genes.

## Introduction

Biallelic mutations in the *OTUD6B* (Ovarian tumor domain-containing 6B) gene were recently described to cause an intellectual disability syndrome characterized by seizures and dysmorphic features in six families worldwide (Santiago-Sim et al., 2017; Online Mendelian Inheritance in Men, OMIM, #617452).

The *OTUD6B* gene (located on chromosome 8q21.3, 17 kb long) encodes a deubiquitinating enzyme (Sobol et al., 2017). Deubiquitinating enzymes (or DUB) participate in regulating different biological processes, such as apoptosis, oncogenes, and tumor suppressor signaling, DNA replication and repair, and checkpoint regulation (Bhattacharya and Ghosh, 2014). Among the approximately 100 DUBs encoded in the human genome, the OTU (ovarian tumor) family comprises at least 16 DUBs containing a complete catalytic triad, including OTUD6B (Xu et al., 2011; Mevissen et al., 2013).

*OTUD6B* shows a widespread expression pattern (The GTEx Portal^1^), and its primary transcript can be alternatively spliced to produce two main different splicing variants. The first isoform (NM_016023.3, OTUD6B-1) is characterized by seven exons with the start codon in the first one, whereas isoform 2 (NM_001286745.1, OTUD6B-2) includes an additional out-of-frame exon (from here named exon 3^∗^) that leads to a premature stop just after three residues. However, the presence of an alternative starting ATG at the 3′ end of exon 3^∗^ leads to the synthesis of a shorter protein lacking the two amino-terminal coiled-coil domains (Mevissen et al., 2013). In non-small cell lung cancer, the two main OTUD6B isoforms have been shown to have opposite effects on global protein synthesis and on DNA synthesis, with OTUD6B-1 stimulating protein and DNA synthesis and OTUD6B-2 repressing both processes (Sobol et al., 2017).

Here, we report the identification of biallelic mutations in *OTUD6B* in a patient with mild intellectual disability associated with seizures and dysmorphic features. We experimentally verified the pathogenic role of the two novel splicing defects, thus providing the first independent replication of the role of *OTUD6B* in this severe multisystem syndrome.

## Materials and Methods

### Patient: The Genetic Odyssey

The proband (normal karyotype 46, XX) was initially submitted to a genetic screening (*CREBBP*, *EP300*) for a suspected Rubinstein–Taybi syndrome (OMIM #180849). Subsequently, she was screened for the two main genes (*KDM6A* and *KMT2D*/*MLL2*) known to be associated with the Kabuki syndrome (OMIM #147920), and later submitted both to a whole-genome CGH-array analysis to define DNA copy number gains and losses and to the analysis of the 22q11.2 locus by FISH. In all cases, no genetic abnormalities were detected. Finally, before whole-exome sequencing (WES), the proband was also screened for a panel of 108 genes responsible for epilepsy. Again, these analyses did not reveal any variant with a clear pathogenic significance.

This study was conducted according to the Declaration of Helsinki and to the Italian legislation on sensible data recording. A signed informed consent for the genetic analysis and for the publication was obtained from the proband’s parents.

### Whole-Exome Sequencing

Genomic DNA was extracted from peripheral blood using an automated DNA extractor Maxwell 16 system (Promega, Madison, WI, United States). WES was performed starting from 50 ng of genomic DNA using the SureSelect Human All Exon V6 Library kit (Agilent Technologies, Santa Clara, CA, United States) and the Illumina NextSeq 500 platform (Illumina, San Diego, CA, United States), according to the manufacturer instructions. Reads were aligned against the human reference genome (hg19) using BWA (Li and Durbin, 2009). The variant calling was performed with SAMtools (Li et al., 2009) and the variant annotation with ANNOVAR^2^ (Wang et al., 2010). Validation of candidate variants and their segregation in the family were confirmed by Sanger sequencing. Primer sequences are available on request.

### Analysis of Splicing Mutations

Total RNA was extracted from peripheral blood mononuclear cells (PBMCs) of the proband, her parents and 5 control individuals, by using the classical phenol-chloroform based protocol (Eurozol, EuroClone, Pero, Italy). Five hundred nanograms of each RNA were reverse-transcribed using the ImProm-II^TM^ Reverse Transcription System (Promega). The effect of the splice variants identified in the proband was evaluated amplifying *OTUD6B* exons 1–4 by RT-PCR (forward primer: 5′-GCATCGCAAAGAGAAGAAGG-3′; reverse primer: 5′-TCTCAAGGCAACCACAGTCA-3′) and analyzing the obtained products by Sanger sequencing. To quantify the aberrant splicing isoforms, a competitive-fluorescent RT-PCR assay was performed using the same primer pair with the forward primer labeled with the 6-FAM fluorophore, essentially as described in Cardamone et al. (2017). Briefly, 1 μl of cDNA was amplified using the GoTaq polymerase (Promega) with a touchdown cycling program (from 67°C to 59°C) for a total of 40 cycles. To minimize the preferential amplification of shorter amplicons, an elongation time of 1 min was used for each cycle. RT-PCR products were resolved by capillary electrophoresis on an ABI-3500DX Genetic Analyzer (Life Technologies, Carlsbad, CA, United States). The relative amount of each splicing product was determined by the GeneMapper v4.0 software (Life Technologies), setting the sum of all measurable peaks equal to 100%. The total *OTUD6B* transcript level was assessed by real-time RT-PCR using the SYBR Premix Ex Taq II (TAKARA Bio USA, Mountain View, CA, United States) on a LightCycler 480 (Roche, Basel, Switzerland). *HMBS* (Hydroxymethylbilane synthase) was used as housekeeping gene.

## Results

### Clinical Report

The proband is a 6-year-old female, second-born of non-consanguineous healthy parents. She was born by cesarean section delivery at 38 weeks of gestation, after a pregnancy complicated by intrauterine growth restriction (IUGR). Her birth weight was 2,095 g. A Tetralogy of Fallot was diagnosed soon after birth, requiring cardiac surgery intervention at the age of 6 months.

During the first 2 months, she showed feeding difficulties, associated with gastroesophageal reflux. Moreover, she also had a neurodevelopmental delay. She sat independently at 11 months, walked at 18 months, and pronounced her first words at 2 years. Due to the occurrence of palpebral ptosis, a Duane syndrome was suspected. Nevertheless, brain MRI was normal, occipitofrontal circumference was 47.8 cm (*z*-score −1.5).

**FIGURE 1:**
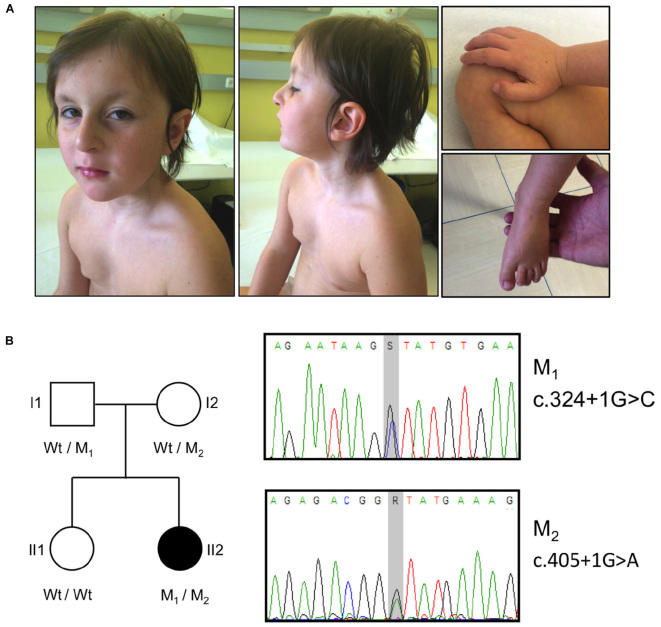
Clinical features, pedigree and mutations in the *OTUD6B* patient. **(A)** Photographs illustrating the phenotype of the patient. The pictures show the facial dysmorphic features (Left and Middle) and the extremities abnormalities (Right). Written informed consent was obtained from the patient’s parents for the publication of images. **(B)** Left: pedigree of the analyzed family. The affected subject is represented by a black symbol. The *OTUD6B* genotype of each individual is indicated below the corresponding symbols. M1: c.324+1G>C, M2: c.405+1G>A; Wt, wild type. Right: sequence chromatograms of the *OTUD6B* regions surrounding the identified mutations. S = G or C, R = G or A.

Main dysmorphic facial features included: large ears, long smooth philtrum, thin upper lip, high arched palate, hanging columella, narrow long face, wide forehead, sunken eyes, and hypotelorism (**Figure 1A**).

The patient also has finger abnormalities, i.e., broad thumbs and first toes, and fetal pads (**Figure 1A**).

At 5 years, tonic-clonic seizures occurred, thus valproate treatment was started. At the latest neurological evaluation, at 6 years of age, she has mild intellectual disability, mild motor difficulty, and episodic behavioral disorders.

The main clinical features of the proband, compared to the ones of previously reported cases, are summarized in **Table 1**.

**Table 1:** Main clinical features of the analyzed patient.

Clinical features	Frequency reported by Santiago-Sim et al. (2017)	Presence in our patient
Intellectual disability	12/12	+
Severe	9/12	
Mild-moderate	3/12	+
Speech delay	9/12	+
Seizure	12/12	+
IUGR	7/12	+
Growth retardation	9/12	
Microcephaly	9/12	+
Hypotonia	9/12	
Feeding difficulties	9/12	+
Gross motor delay	9/12	
Structural brain abnormalities	6/12	
Congenital heart defects	4/12	+^∗^
Progressive scoliosis	5/12	
Large/protruding ears	8/12	+
Long philtrum	7/12	+
Thin upper lip	6/12	+
Long palpebral fissures	6/12	
High arched palate	5/12	+
Prominent/high nasal bridge	5/12	
Retrognathia	4/12	
Arched eyebrows	3/12	
Abnormalities of fingers and/or toes	11/12	+
Broad thumbs	6/12	+
Overriding toes	3/12	
Fetal pads	1/12	+

*Our patient’s clinical features are compared with those already described in patients with biallelic OTUD6B mutations. ^∗^Tetralogy of Fallot. IUGR, Intrauterine growth restriction.*

### WES Identified Two Putative Variants in *OTUD6B*

Whole-exome sequencing was performed on the proband’s DNA with a mean coverage depth of 110× and 98% of the bases of the target region covered at ≥20×.

Data analysis highlighted the presence of two heterozygous variants affecting exons 2 (c.324+1G>C) and 3 (c.405+1G>A) donor splice sites of the *OTUD6B* gene (NM_016023.3). These variants (rs767665903 and rs751309307) are extremely rare in the general population, with a frequency in the genome aggregation database (GnomAD)^3^ (Lek et al., 2016) of 4.18 × 10^−6^ and 9.87 × 10^−6^, respectively. The segregation in the family was confirmed by Sanger sequencing: the father (I1) was carrier of the c.324+1G>C mutation whereas the mother (I2) was heterozygous for c.405+1G>A. The proband’s sister (II1) was wild type for both variants (**Figure 1B**).

### Splicing Variant Characterization

**FIGURE 2:**
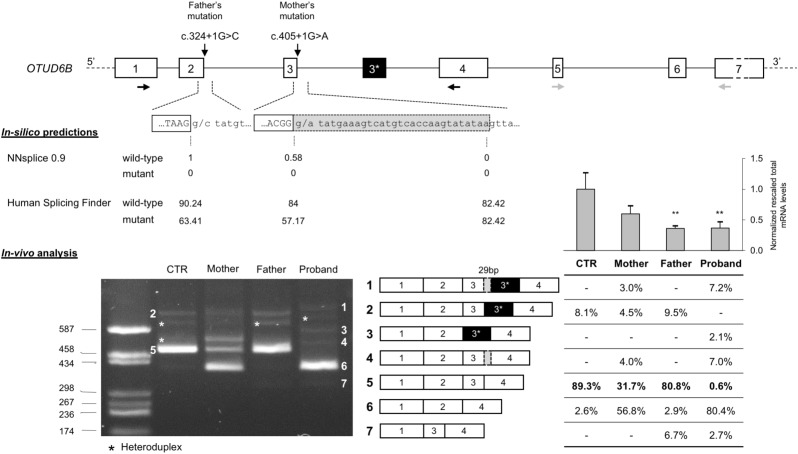
Molecular characterization of c.324+1G>C and c.405+1G>A splicing mutations. **(Top)** Schematic representation of the *OTUD6B* genomic region: introns are represented by lines, whereas exons (approximately drawn to scale) are shown as boxes (in black, the alternative exon 3^∗^ of isoform 2; in light gray the 29-bp inclusion of the aberrant transcript); the identified mutations are also indicated, RT-PCR primers (1F and 4R), in black, and real-time RT-PCR primers (5F and 7R), in gray, are drawn as horizontal arrows. **(Middle)** *In silico* predictions of the effect of c.324+1G>C and c.405+1G>A mutations on splicing; the scores calculated by the NNSPLICE 0.9 and Human Splicing Finder tools are reported below each splice site. **(Bottom)** Left: agarose gel (2%) showing the results of the RT-PCR assay (1F-4R) performed on the RNA extracted from the PBMCs of the patient, the parents, and a control individual (CTR). All the obtained bands have been eluted from the gel and submitted to Sanger sequencing. The aberrant and wild-type splicing events observed in the patient and in her parents are schematically represented. Right: the table shows the quantitation of each PCR product calculated using the fluorescent competitive RT-PCR assay (using the same PCR conditions as for the gel electrophoresis analysis); above the table, bars represent the total *OTUD6B* transcript level measured by real-time RT-PCR on the RNAs of all family members and five controls; data are presented as normalized rescaled values and were analyzed by *t*-test. ^∗∗^*p* < 0.01.

The effect of the identified mutations on splicing was first evaluated *in silico*, using two different software: NNSPLICE 0.9 and Human Splicing Finder. The NNSPLICE algorithm predicted that both mutations completely abolish the corresponding donor splice site. The Human Splicing Finder software confirmed the deleterious effects of these variants; in particular, concerning exon 2 donor site, the score decreases from 90.24 to 63.41, whereas, for exon 3, from 84 to 57.17 (**Figure 2**).

We confirmed these predictions evaluating by RT-PCR the possible aberrant splicing directly on the patient RNA and, to better discriminate the effect of the two variants, we also examined the parents’ RNA. Agarose gel electrophoresis of the obtained RT-PCR products showed that both variants affect *OTUD6B* splicing and lead to the production of aberrant transcripts (**Figure 2**). In detail, c.324+1G>C mutation mainly causes the complete skipping of exon 2 that leads to a frame-shift and, consequently, to the introduction of a premature stop codon after six amino acids of the mutated protein (p.Ala58Aspfs^∗^6). This alternative transcript is likely to be degraded by the nonsense-mediated mRNA decay mechanism (NMD) (Maquat, 2005) as demonstrated by the extremely low intensity of the corresponding amplicon in the father and in the proband RT-PCR (band 7, **Figure 2**).

Regarding the c.405+1G>A variant, the major alternative splicing event observed was exon-3 skipping (band 6, **Figure 2**), but the RT-PCR performed on the mother’s and on the proband’s RNA also showed the presence of a second aberrant transcript corresponding to the inclusion of a portion of intron 3, due to the activation of a cryptic donor splice site located 29 bp downstream of the canonical one (band 4, **Figure 2**).

Moreover, in the RNA samples of the proband’s parents and of the control subject we confirmed the existence of a physiological alternative transcript (band 2, **Figure 2**) characterized by the inclusion of a 140-bp pseudoexon (exon 3^∗^). In the proband, as expected, we observed two alternative versions of this isoform: one lacking exon 3 (band 3, **Figure 2**) and the other including the 29-bp longer version of exon 3 (band 1, **Figure 2**).

Furthermore, we performed quantitative analysis by competitive-fluorescent RT-PCR showing that the proband presents less than 1% of the wild-type transcript, further strengthening the causative role of the identified splicing variants (**Figure 2**).

Finally, we measured the total *OTUD6B* transcript levels by real-time RT-PCR on the parents’ and proband’s RNAs and on five control subjects. The obtained results confirmed that the two individuals carrying the c.324+1G>C mutation present a significant reduction of the mRNA levels compatible with the degradation by the NMD pathway of the transcripts lacking exon 2 (**Figure 2**).

## Discussion

Here, we used WES – together with functional studies – to provide a child with mild intellectual disability, seizures, and dysmorphic features with a molecular diagnosis, after multiple inconclusive panel-based and phenotype-driven genetic screenings.

Two heterozygous splicing mutations were found in *OTUD6B*, a gene to which no genetic diseases had been associated when we completed exome data analysis (March 2017). A fundamental help in understanding the causative role of the identified mutations came from the almost coincident publication of a paper reporting, for the first time, biallelic mutations in *OTUD6B* in patients with a phenotype strikingly similar to our patient (Santiago-Sim et al., 2017). We confirmed the causative role of the identified mutations characterizing their effect on *OTUD6B* splicing directly on the patient’s RNA, taking advantage of the widespread expression of the gene (The GTEx Portal; see text footnote 1).

The six previously described families with mutations in *OTUD6B* showed a wide spectrum of clinical manifestations (Santiago-Sim et al., 2017); our patient, even if she is carrier of two severe splicing mutations, presents a milder phenotype compared to the previously described siblings with a homozygous splicing mutation affecting exon 2 (Family 5 in the Santiago-Sim paper). Unfortunately, the authors did not experimentally validate the effect of the c.173-2A>G mutation on splicing, so we can only speculate that the less severe phenotype we observed in our proband might be due to the small amount of functional protein produced by the residual wild-type splicing, which we could estimate, by fluorescent RT-PCR assays, to be about 0.6% of the total. Another possibility to explain the milder phenotype of our patient could lay in the partial functionality of the OTUD6B protein synthesized from the maternal mutated transcripts. In fact, the aberrant splicing produced by the c.405+1G>A mutation is in frame, allowing the production of an incomplete protein, lacking 27 amino acids. The mutant protein is predicted to be devoid of a portion of the N-terminal coiled-coil domain.

It is interesting to note that exon-3 skipping was also found in the wild-type sample (2.6% of the total), suggesting that this skipping is a naturally occurring alternative splicing of *OTUD6B* (**Figure 2**). The predicted protein would be 3 kDa heavier than the OTUD6B-2 isoform. Indeed, in western blots reported by Sobol et al. (2017), who characterized the different protein isoforms, a band above the OTUD6B-2 one is clearly visible and could well correspond to the exon-3 lacking isoform. The exon-3 lacking transcript amounts to the 80.8% of total *OTUD6B* mRNAs in the proband, while exon-2 skipped mRNAs only represent the 2.6%, suggesting that transcripts originating from the paternal allele are significantly downregulated by the NMD pathway. This hypothesis is further confirmed by the results obtained measuring the total *OTUD6B* transcript levels on all family members (**Figure 2**).

Considering the existence of two protein isoforms with different functions, it is also interesting to evaluate the impact of mutations in *OTUD6B* on both protein variants. In this frame, our quantitative analysis of splicing isoforms showed that the proband and both of her parents had similar amounts of transcripts that could probably produce a wild-type OTUD6B-2 protein compared to the control individual (9.3% in the proband, 9.5% in the father, 7.5% in the mother vs. 8.1% in the control; isoforms 1+2+3 in **Figure 2**). One can therefore speculate that the mild phenotype of our patient could be at least partially due to the fact that both her mutations do not affect the short OTUD6B-2 protein variant, at difference with what found in previously reported *OTUD6B* families. In fact, five out of six families described by Santiago-Sim et al. (2017) had mutations affecting both isoforms, with only Family 5, bearing a mutation (c.173-2A>G) potentially compatible with the production of an intact OTUD6B-2 protein. However, the skipping of exon 2, the most likely consequence of c.173-2A>G, would cause a severe degradation of the corresponding transcript by NMD, as confirmed by the observation that transcripts simultaneously lacking exon 2 and containing exon 3^∗^ were undetectable in the RNA extracted from the father of our patient (**Figure 2**).

## Conclusion

We identified two novel heterozygous *OTUD6B* splicing mutations (c.324+1G>C and c.405+1G>A) in an Italian child with an intellectual disability syndrome, reinforcing the concept that mutations in genes encoding enzymes involved in regulating post-translational modifications play an important role in intellectual disability and related syndromes (Bustos et al., 2018). The qualitative and quantitative analysis of the consequences of the identified mutations on splicing was instrumental to understand the contribution of the two alleles to disease pathogenesis, an essential prerequisite to set up future personalized therapies (i.e., small molecules, modified snRNAs, or antisense oligonucleotides) (Slaugenhaupt et al., 2004; Hammond and Wood, 2011; Donadon et al., 2018) with the aim to directly correct the molecular defects. The application of exome-sequencing once more revealed to be a straightforward approach to the genetic diagnosis in a case with a complex phenotype partially overlapping with different syndromes and suggests that, in case of suspected Rubinstein–Taybi syndrome with negative results for mutations in *CREBBP* and *EP300* genes, the screening of *OTUD6B* should be performed and its associated syndrome (intellectual developmental disorder with dysmorphic facies, seizures, and distal limb anomalies; IDDFSDA, OMIM#617452) should be included as a differential diagnosis.

## Author Contributions

LS and RA conceived and designed the experiments. VR and LS performed the experiments. GS and LS analyzed the exome data. LS drafted the paper. MB and GB clinically evaluated and followed the patient. SD and RA critically revised the manuscript. SD supervised the entire study.

## Conflict of Interest Statement

The authors declare that the research was conducted in the absence of any commercial or financial relationships that could be construed as a potential conflict of interest.

## Footnotes

1 https://www.gtexportal.org/home/

2 http://annovar.openbioinformatics.org/en/latest/

3 http://gnomad.broadinstitute.org/, accessed August 3, 2018.
